# *SEPT9* Gene Methylation as a Noninvasive Marker for Hepatocellular Carcinoma

**DOI:** 10.1155/2020/6289063

**Published:** 2020-10-29

**Authors:** Baoliang Li, Hao Huang, Ronghai Huang, Wei Zhang, Guangpeng Zhou, Zhen Wu, Chunhua Lv, Xiaoliang Han, Li Jiang, Yongjun Li, Baohua Li, Zhongtao Zhang

**Affiliations:** ^1^Department of General Surgery, Beijing Friendship Hospital, Capital Medical University, Beijing Key Laboratory of Cancer Invasion and Metastasis Research and National Clinical Research Centre for Digestive Diseases Beijing 100050, China; ^2^Department of General Surgery, Beijing Ditan Hospital, Capital Medical University, Beijing 100015, China; ^3^BioChain (Beijing) Science & Technology Inc., Beijing 102600, China; ^4^Department of Respiratory Medicine, Mentougou District Hospital of Beijing, Beijing 100230, China

## Abstract

**Background:**

Early detection appears to be the most effective approach to improve the overall survival of patients with hepatocellular carcinoma (HCC). We evaluated the potential performance of plasma *SEPT9* methylation (mSEPT9) as a noninvasive biomarker for the diagnosis of patients with HCC.

**Methods:**

A total of 373 subjects were included, and the group consisted of 104 HCC patients, 95 with an at-risk disease, and 174 healthy controls (HC). The methylation of mSEPT9 was determined using methylation-specific fluorescence quantitative PCR. The diagnostic performance of plasma mSEPT9 for HCC was assessed in a single-blind manner.

**Results:**

The receiver operating characteristic (ROC) curve showed that plasma mSEPT9 can be used to detect and discriminate HCC with an area under the ROC curve (AUROC) of 0.961, a sensitivity of 82.7%, and specificity of 96.0% from HC. These results showed that plasma mSEPT9 had better diagnostic performance than serum alpha fetoprotein (AFP) (AUROC 0.881, sensitivity 57.7%, and specificity 98.3%). Similar results were noted in the detection of early-stage HCC. When combined with serum AFP, the sensitivity increased to 91.3% and 87.7% for the detection of HCC and early-stage HCC,respectively. Notably, the levels of plasma mSEPT9 dramatically decreased after surgery (*P* = 0.001).

**Conclusions:**

Plasma *SEPT9* methylation might serve as a useful and noninvasive biomarker for the diagnosis of HCC and can be used to evaluate the therapeutic efficacy of HCC treatment.

## 1. Introduction

Liver cancer is currently the sixth most common malignant disease and the fourth leading cause of cancer death worldwide with approximately 841,000 new cases and 782,000 deaths annually [1]. In China, the incidence and mortality of liver cancer ranks fourth and second, respectively, in newly diagnosed cancer cases and cancer deaths, which accounts for approximately half of the new cases and deaths worldwide [2]. Hepatocellular carcinoma (HCC) is the most common form of liver cancer, and it is closely linked to cirrhosis, chronic infection with hepatitis B virus (HBV) or hepatitis C virus (HCV), and metabolic and alcoholic liver disease [3]. Despite improvements in the diagnosis and treatment of this cancer, the prognosis and survival remain very poor, with a five-year survival rate of less than 30%. This rate is primarily due to it being diagnosed at a late stage and the missed opportunity to receive optimal therapy for HCC patients [4]. Early-stage detection and receipt of curative therapy appear to be the most effective approach to improve the overall survival of HCC patients [5].

Currently, the serum alpha fetoprotein (AFP) assay and ultrasonography (US) are the primary methods used to detect and diagnose HCC [6]. However, AFP has been questioned due to its low sensitivity (25–65%) at the commonly used cut-off value of 20 ng/mL [7]. In addition, US sensitivity for early-stage HCC appears similar to that of serum AFP and relies largely on the presence of cirrhosis, the size of the tumor, and equipment and examiner expertise. Therefore, serum AFP and US are not satisfactory methods for the early diagnosis of HCC, and new effective biomarkers are urgently needed.

It has been recently reported that HCC is characterized by multiple genetic and epigenetic changes [8]. In contrast to genetic modifications, epigenetic modifications do not change the DNA sequence, but they change the DNA conformation and expression, which plays an important role in tumorigenesis and progression [9]. DNA hypermethylation is an epigenetic modification and an early cancer event that can be detected in plasma-derived cell-free DNA, making it an ideal and useful biomarker for early detection and staging of cancer [10]. Many studies have demonstrated that DNA methylation analysis of specific genes, such as *p16*, *p15*, *RASSF1A*, *APC*, and *SOCS1*, can be applied for the early detection and diagnosis of HCC patients [10, 11]. However, to date, no circulating epigenetic biomarker has been approved to diagnose HCC at the individual level.

The *septin9* gene (*SEPT9*) methylation test is the first blood-based methylation test approved by the United State Food and Drug Administration (US FDA) as a colorectal cancer screening test [12]. Previous work has suggested that *SEPT9* plays a key role in cell division and is a candidate tumor suppressor gene whose hypermethylation is associated with carcinogenesis [13, 14]. *SEPT9* expression is frequently silenced via aberrant promoter hypermethylation in liver cancer, and it is a significant epidriver gene in liver carcinogenesis [15]. Recently, it has been reported that among patients with cirrhosis, the *SEPT9* methylation (mSEPT9) test constitutes a promising circulating epigenetic biomarker for HCC diagnosis at the individual patient level [16]. Given that the underlying causes of liver cancer in Chinese populations are different from those in Europe, this study was designed to investigate whether *SEPT9* methylation can be detected in the plasma of HCC patients in Chinese populations and subsequently be used as a noninvasive marker for HCC detection.

## 2. Materials and Methods

### 2.1. Study Design and Patient Selection

In this study, patients with signs and symptoms of liver malignancy, including 104 hepatocellular carcinoma (HCC) patients, 95 with at-risk liver disease (cirrhosis, hepatitis, and focal nodular hyperplasia), and 174 healthy controls (HC) who had been deemed healthy after undergoing a physical examination from Beijing Ditan Hospital Capital Medical University, were enrolled from July 2018 to January 2019. All of the participants provided plasma samples, and the mSEPT levels were measured in a blinded manner. The final diagnostic result was based on at least two imaging methods (ultrasound, CT, and MRI) and a histopathological confirmation. Patients who had coexisting tumors, metastatic carcinoma, or an undefined pathological status were excluded. Plasma samples were also collected from 15 HCC patients prior to and 10 days after tumor resection to evaluate the postsurgical prognostic value of plasma mSEPT9. All of the study subjects, including 199 patients and 174 healthy controls, underwent simultaneous detection for AFP. The normal range for AFP in this study was determined to be 0–20 ng/mL, and values greater than 20 were considered abnormal. This study was approved by the Beijing Ditan Hospital Capital Medical University Ethics Committee, and all of the subjects received informed consent.

### 2.2. Sample Collection and Storage

Standard operating protocols and blood collections were performed as previously described [17]. A 10 mL venous blood sample was collected from each subject using K_2_EDTA anticoagulant tubes (BD Biosciences, USA). Plasma was isolated within four hours post blood collection using centrifugation at room temperature (1350 g, 12 min). Plasma samples were centrifuged again (1350 g, 12 min) to remove residual blood cells. The storage and transportation of samples was conducted following the user manual of the Diagnostic Kit for Septin9 Gene Methylation Assay (BioChain (Beijing) Science and Technology, Inc., Beijing, P.R. China). Sample quality was assessed as previously described [18].

### 2.3. Quantitative Methylation-Specific PCR (qMSP) for SEPT9 Assay

DNA extraction and bisulfite conversion were performed manually following the manufacturer's instructions for the Diagnostic Kit for *Septin9* Gene Methylation Assay (BioChain (Beijing) Science and Technology, Inc., Beijing, P.R. China). Methylated target sequences in the bisulfite-treated DNA template were amplified using real-time PCR on an ABI7500 PCR device (Life Technologies). PCR was performed in a single 60 *μ*L PCR with 25 *μ*L of template DNA per well and run through the reaction system. This process consisted of an initial 30-minute activation at 94°C, followed by 45 cycles at 62°C for 5 seconds, 55.5°C for 35 seconds, and 93°C for 30 seconds, and then cooling at 40°C for 5 seconds. In the quantitative methylation-specific PCR, *β-actin* (*ACTB*) was used as an internal control to assess the quantity of the input DNA and the validity of the PCR amplification. The validity of each sample batch was determined on the basis of the methylated *SEPT9* and *ACTB* cycle threshold (CT) values for the positive and negative controls. Positive and negative controls were provided in the kit and run in parallel with the samples each time. The detection threshold and the reaction factors were examined and optimized using clinical plasma samples.

### 2.4. Statistical Analysis

The statistical analysis was performed using the SPSS 20.0 software (IBM Corporation, Armonk, NY), and graphs were made using GraphPad Prism 5.0 for Windows. The differences in the CT value of the plasma SEPT9 methylation assay between different groups were analyzed using Student's *t* test. The association between the plasma mSEPT9 status of the HCC patients and their clinicopathological parameters was analyzed using Fisher's test. To assess the diagnostic performance of *SEPT9* gene methylation either alone or in combination with AFP between HCC or early-stage HCC and HC or at-risk disease, the models were assessed using logistic regression. After this, the new variable predicted probability (*P*) was subjected to receiver operating characteristic curve (ROC) analysis. The ROC was generated to calculate the respective areas under the curves (AUCs) with a 95% confidence interval (CI), sensitivity, specificity, positive predictive value (PPV), negative predictive value (NPV), and accuracy of the SEPT9 gene methylation either alone or in combination with AFP for HCC diagnosis at the determined cut-off value. Differences in the efficacy evaluation for HCC patients were evaluated using a paired *t* test. A two-tailed probability of *P* < 0.05 was considered to be statistically significant.

## 3. Results

### 3.1. Methylation Status of SEPT9 in Plasma DNA within Different Groups

**Figure 1:**
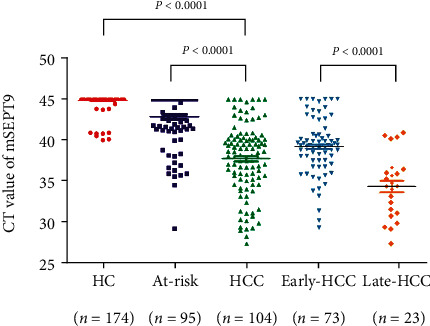
The plasma SEPT9 methylation (mSEPT9) level in cell-free DNA of plasma assayed using methylation-specific fluorescence quantitative PCR. Quantitative PCR showed a significantly decreased median CT value of SEPT9 methylation assay in hepatocellular carcinoma (HCC, *n* = 104) when compared to the healthy control (HC, *n* = 174) and at-risk disease (*n* = 95). The at-risk disease category indicates noncancerous liver diseases, including cirrhosis and hepatitis. CT indicates cycle threshold. Early-HCC represents patients with early-stage (stages A and B) HCC. Late-HCC represents patients with late-stage (stages C and D) HCC. The full line shows the mean CT of each group.

The *SEPT9* promoter methylation (mSEPT9) status in plasma from 104 patients with HCC, 95 patients with at-risk disease, and 174 healthy controls (HC) was assayed. The baseline characteristics of the participants are shown in Table 1. Since the baseline characteristics were not detected using the assay for most normal controls, the cycle threshold (CT) value was set at 45 (the maximal number of PCR cycles in the assay) for those not detected samples. The CT value of mSEPT9 in the HCC patients was significantly lower than in the HCs (HCC vs. HCs, *P* < 0.0001) and in the at-risk disease patients (HCC vs. at-risk, *P* < 0.0001), suggesting elevated levels of mSEPT9 in the plasma of HCC patients (Figure 1 and Table S1). We also analyzed the same data with the Wilcoxon-Mann-Whitney test and got the same conclusion (Figure S1).

### 3.2. Diagnostic Value of Plasma mSEPT9 in Patients with HCC and Early-Stage HCC

**Figure 2:**
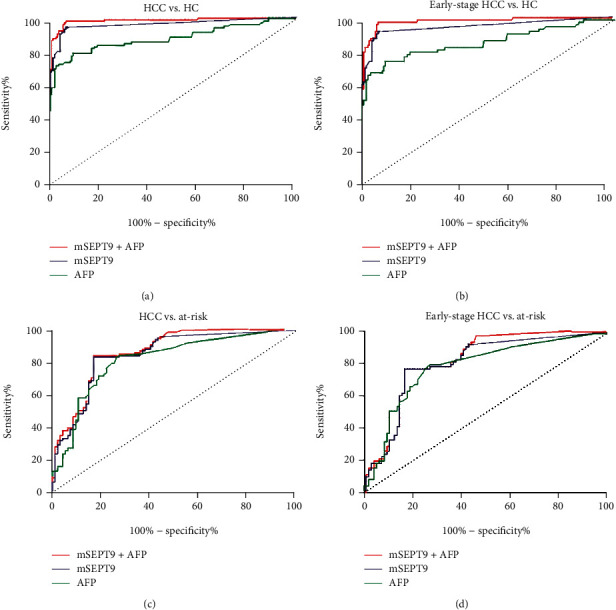
The receiver operating characteristic (ROC) curve of plasma SEPT9 methylation (mSEPT9) and serum alpha fetoprotein (AFP) in the diagnosis of HCC and early-stage HCC from the healthy control group and the at-risk disease group. The ROC curves of plasma mSEPT9 with and without the inclusion of AFP and AFP alone for HCC patients versus HC (a), early-stage HCC patients versus HC (b), HCC patients versus at-risk disease (c), and early-stage HCC patients versus at-risk disease (d). HCC indicates hepatocellular carcinoma. HC indicates healthy control. At-risk indicates noncancerous liver diseases including cirrhosis and hepatitis.

To achieve the observed test performance, ROC curve analysis was used to evaluate the area under the curve (AUC), sensitivity, and specificity and to determine the best cut-off CT value of mSEPT9 for HCC diagnosis. Compared with the HC, AUC was estimated to be 0.961 (95% CI 0.933–0.989), and mSEPT9 had a sensitivity of 82.7% (86/104) and a specificity of 96.0% (167/174) at the CT cut-off value of 41. In addition, the positive predictive value (PPV), negative predictive value (NPV), and accuracy were 92.5%, 90.3%, and 91.0%, respectively (Figure 2(a) and Table 2). Similar results were obtained from a comparison between patients with HCC and those with at-risk diseases. AUC was estimated to be 0.843 (95% CI 0.787–0.899), and mSEPT9 had a sensitivity of 82.7% (86/104) and a specificity of 83.2% (79/95) at the CT cut-off value of 41. In addition, the PPV, NPV, and accuracy were 84.3%, 81.4%, and 82.9%, respectively (Figure 2(c) and Table 2).

The CT value of plasma mSEPT9 in patients with late-stage HCC (stages C/D) was significantly decreased than those in patients with early-stage HCC (stages A/B) (*P* < 0.001, Figure 1), which indicated that plasma mSEPT9 positively correlated with HCC malignancy. The ROC curve showed that plasma mSEPT9 demonstrated a remarkable performance in the diagnosis of patients with early-stage HCC as compared to the HC participants (AUC 0.946, 95% CI 0.907–0.985, sensitivity 76.7%, and specificity 96.0%), and at-risk disease (AUC 0.799, 95% CI 0.730–0.868, sensitivity 76.7%, and specificity 83.2%) (Figures 2(b) and 2(d) and Table 2). All of these results demonstrate that plasma mSEPT9 can be used as an excellent biomarker for the diagnosis of HCC and detection of early-stage HCC in clinical settings.

### 3.3. The Diagnostic Value of Combining Serum AFP Concentration and Plasma mSEPT9 in Patients with HCC and Early-Stage HCC

Serum AFP is the most accepted and used biomarker for HCC detection and diagnosis. In this study, the differential diagnosis performances of plasma mSEPT9, AFP, and their combination were evaluated. It was shown that the use of serum AFP (AUC 0.881, 95% CI 0.833–0.928, sensitivity 57.7%, and specificity 98.3%), plasma mSEPT9 (AUC 0.961, 95% CI 0.933–0.989, sensitivity 82.7%, and specificity 96.0%), or their combination (AUC 0.986, 95% CI 0.974–0.999, sensitivity 91.3%, and specificity 94.8%) significantly improved the diagnostic ability of HCC in healthy individuals (Figure 2(a) and Table 2). The diagnosis between HCC and those with at-risk diseases also showed that plasma mSEPT9 and the combination were more sensitive than AFP alone (Figure 2(c) and Table 2).

Similar results were also obtained using a comparison between patients with early-stage, HC controls, and those with at-risk diseases. At a cut-off of 20 ng/mL, AFP had an AUC (0.852, 95% CI 0.789–0.914) sensitivity of 52.1% and specificity of 98.3% in distinguishing early-stage HCC from HC. The combination plasma mSEPT9 with serum AFP improved AUC (0.980, 95% CI 0.962–0.999) and sensitivity (87.7%) and lessened the decreased diagnostic specificity (94.8%) (Figure 2(b) and Table 2). The diagnosis between early-stage HCC and those at-risk for the disease also showed that the plasma mSEPT9 and the combination were more sensitive than AFP alone (Figure 2(d) and Table 2).

**Figure 3:**
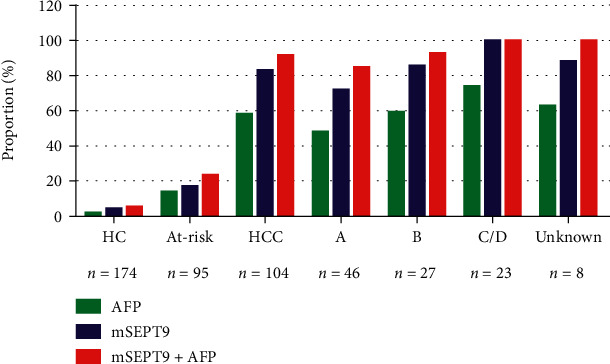
Positive detection rate of plasma SEPT9 methylation (mSEPT9) and serum AFP for controls and at all stages of HCC. HC indicates healthy control. At-risk indicates noncancerous liver diseases including cirrhosis and hepatitis. HCC indicates hepatocellular carcinoma (a–d), and unknown indicates stages of HCC according to the Barcelona Clinic Liver Cancer (BCLC) stage system. AFP means alpha fetoprotein.

**Figure 4:**
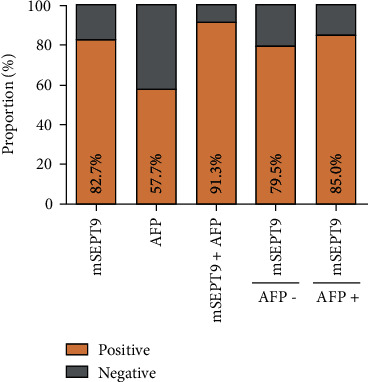
The positive rate for mSEPT9, serum AFP, or the combination by AFP status in HCC. The positive rates of mSEPT9 diagnosis in AFP-negative and AFP-positive HCC patients were comparable (79.5% vs. 85.0%, *P* = 0.468), which indicated the diagnosis of mSEPT9 in HCC irrespective of AFP status. HCC indicates hepatocellular carcinoma. AFP means alpha fetoprotein.

The positive detection rate of plasma mSEPT9 and serum AFP for controls and each HCC stage are presented as a histogram in Figure 3. There was a trend that plasma mSEPT9 assay detected more late-stage HCC cases than early-stage HCC cases (Figure 3). Combining the assay of plasma mSEPT9 test with serum AFP enhanced the test sensitivity in HCC detection. These results suggested that plasma mSEPT9 assay exhibited high sensitivity for HCC at each stage in opportunistic screening. When considering AFP status, it was found that the positive rates of mSEPT9 diagnosis in the AFP-negative and AFP-positive HCC patients were comparable (79.5% vs. 85.0%, *P* = 0.468) (Figure 4), which indicated the diagnosis of mSEPT9 in HCC cases irrespective of the AFP status. These results demonstrated that plasma mSEPT9 can complement the diagnosis of AFP-negative HCC and AFP-limited HCC patients with a remarkable discriminating performance.

### 3.4. Correlation of Plasma mSEPT9 with the Clinicopathological Characteristics of Patients with HCC

The association of plasma mSEPT9 and the clinicopathological characteristics of the patients with HCC are shown in Table S2. Plasma mSEPT9 was not corrected for gender, serum AFP level, or HBeAg status. Plasma mSEPT9 was more frequently observed in older patients (>50) and with late-stage HCC than in those who were younger (*P* = 0.002) and with early-stage disease (*P* = 0.03).

### 3.5. Plasma mSEPT9 Can Monitor the Efficacy of Treatment in Patients with HCC

**Figure 5:**
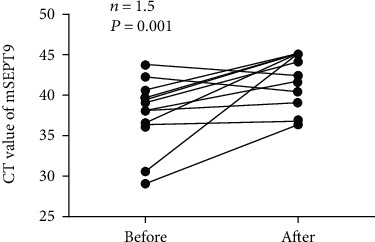
The mSEPT9 levels (CT values) for HCC patients before and 10 days after surgery. Before and After represent before surgery and after surgery, respectively. HCC indicates hepatocellular carcinoma. CT means cycle threshold.

Dynamic changes in biomarkers that reflect a patient's condition can provide clinical guidance for doctors. In this study, the plasma mSEPT9 levels (CT values) in paired plasma samples from 15 cases undergoing surgery were assessed. The mean CT value of plasma mSEPT9 was 37.9 (range: 29.1–43.7) before surgery and 42.2 (range: 36.3–45.0) after 10 days (*n* = 15, *P* = 0.001) (Figure 5). Moreover, the CT plasma mSEPT9 values in all 13 patients with positive mSEPT9 (CT<41) before surgery decreased dramatically after 10 days. Therefore, dynamic changes in the plasma mSEPT9 levels of HCC patients can be used to monitor the efficacy of treatment.

## 4. Discussion

In the diagnosis of HCC, plasma mSEPT9 showed remarkable sensitivity and specificity for detection in HCC patients, even in the early stages from a healthy individual and those with at-risk diseases. Combined with serum AFP, plasma mSEPT9 showed significantly superior sensitivity than AFP alone in distinguishing HCC patients from both controls. Plasma mSEPT9 can be used to compliment the diagnosis of AFP-negative HCC and AFP-limited HCC patients with a remarkable discriminating performance. Moreover, dynamic changes in the plasma mSEPT9 levels in HCC patients can be used to monitor the efficacy of treatment. These findings showed that plasma mSEPT9 can be used as a noninvasive diagnosis biomarker for HCC patients.

DNA hypermethylation is an important event in carcinogenesis that can be detected in plasma-derived cell-free DNA, which makes it an ideal and useful biomarker for early detection and staging of cancer. Many studies have demonstrated that DNA methylation analysis of specific genes can be used for the early diagnosis of cancer, such as plasma mSEPT9 in colorectal cancer [19], bronchoalveolar lavage fluid *SHOX2* and *RASSF1A* in lung cancer, [20], and urine *GSTP1* in prostate cancer [21]. However, there is no FDA-approved circulating epigenetic biomarker that has been shown to be useful to diagnose HCC.

Septins are an evolutionarily conserved family of GTPases with diverse functions that include roles in cytokinesis, vesicle trafficking, apoptosis, and the maintenance of cell polarity [22]. *SEPT9*, a member of the septin family, acts as a tumor suppressor gene in carcinogenesis and is silenced or decreased by aberrant promoter methylation, which can be reactivated by treatment with azacytidine, providing evidence of epigenetic mechanisms of this gene during carcinogenesis [14, 23]. Several studies have suggested that *SEPT9* acts a tumor suppressor gene and plays an important role in the regulation of cell division during its hypermethylation in liver carcinogenesis [15, 24]. It has been recently reported that plasma mSEPT9, as a novel circulating cell-free DNA-based epigenetic biomarker, can be used to diagnose HCC at the individual patient level with 80.8% sensitivity and 95.8% specificity [16]. In Europe, the etiology of cirrhosis and HCC is primarily hepatitis C and alcoholic cirrhosis, while in China, the focus is primarily on hepatitis B. In this study, the diagnostic performance of plasma mSEPT9 in a Chinese population [25] was studied. It was found that the diagnostic performance of plasma mSEPT9 was in accordance with a previous study conducted in Europe. Plasma mSEPT9 exhibited a high diagnostic performance for HCC, and thus, it could be considered a promising biomarker for diagnosing HCC among healthy individuals and patients with at-risk diseases.

HCC is the most common type of primary liver cancer that is usually diagnosed at advanced stages due to a lack of effective diagnostic biomarkers. Serum AFP is the most widely used biomarker for the diagnosis of HCC. However, the serum AFP level lacks adequate sensitivity and specificity for early diagnosis of HCC, with nearly one-third of early-stage HCC patients testing negative for AFP [7]. Therefore, AFP is not a satisfactory biomarker for early diagnosis and prognostic evaluation. In this study, the differential diagnosis performances of plasma mSEPT9, AFP, and their combination were evaluated. It was shown that, in comparison with serum AFP, plasma mSEPT9 or the combination significantly improved the diagnostic ability between HCC and healthy individuals and between HCC and those with at-risk diseases. Plasma mSEPT9 complemented the diagnosis of AFP-negative HCC and AFP-limited HCC patients with a remarkable discriminating performance. In addition, dynamic changes of plasma mSEPT9 in HCC patients can be used to monitor treatment efficacy. Therefore, it is conceivable that plasma mSEPT9 is a promising noninvasive diagnostic biomarker for HCC patients.

The plasma mSEPT9 assay was shown to be an excellent test for HCC detection in a previous study [16]. The application of three PCR reactions and the specific probe for mSEPT9 maximized the opportunity to detect a trace amount of methylated *SEPT9* DNA. However, in this study, kits were made using BioChain to conduct the single-tube PCR with double the volume. This reduced the number of tests and the chance of contamination and lowered the costs of the assay. This method will benefit patients in future screenings. This simplified mSEPT9 assay reduced the complexity of the test process from three PCR reactions to one, which could potentially benefit many patients without reducing the detection performance. Hence, this is a simpler, cheaper, more efficient, and convenient method for HCC screening.

Although the plasma mSEPT9 displayed a good performance for HCC detection among healthy individuals and patients with at-risk diseases, it also exhibited a cross-specificity with colorectal cancer. Nevertheless, patients with at-risk diseases, such as those with cirrhosis, are more likely to develop HCC than any other type of cancer [26]. In addition, the positive rate of plasma mSEPT9 (4.1%) in the screening population and the prevalence of colorectal cancer was low, which resulted in a dramatically reduced positive predictive value (PPV). Thus, a positive plasma mSEPT9 test in a patient with an at-risk disease in the liver should initially evoke a high level of suspicion for HCC instead of colorectal cancer. Multicenter, large-scale cohort studies of patients would be helpful for evaluating the diagnostic accuracy of the plasma mSEPT9 test in screening settings.

## 5. Conclusions

This study demonstrated that plasma mSEPT9 can be a promising noninvasive and circulating epigenetic biomarker for HCC diagnosis at the individual level. In addition, the combination of plasma mSEPT9 and AFP could enhance the sensitivity of AFP alone in the diagnosis of HCC.

## Figures and Tables

**Table 1:** Baseline characteristics of the participants.

	HCC (%)	At-risk (%)	HC (%)	Statistics *χ*^2^	*P* value
*N*	104	95	174		
Gender					
Male	83 (79.8)	56 (58.9)	100 (57.5)	15.562	<0.001
Female	21 (20.2)	39 (41.1)	74 (42.5)		
Age					
0-50	33 (31.7)	36 (37.9)	83 (47.7)	7.307	0.026
>50	71 (68.3)	59 (62.1)	91 (52.3)		
Cirrhosis					
Yes	80 (76.9)	63 (66.3)	NA	2.762	0.097
No	24 (23.1)	32 (33.7)	NA		
Etiology					
HBV	89 (85.6)	59 (62.1)	NA	23.353	<0.001
HCV	10 (9.6)	7 (7.4)	NA		
Alcoholic	2 (1.9)	8 (8.4)	NA		
Others	3 (2.9)	21 (22.1)	NA		
AFP					
0-20 ng/mL	44 (42.3)	82 (86.3)	171 (98.3)	129.220	<0.001
>20 ng/mL	60 (57.7)	13 (13.7)	3 (1.7)		
BCLC stage					
A	46 (44.2)				
B	27 (26.0)				
C/D	23 (22.1)				
Unknown	8 (7.7)				

HCC indicates hepatocellular carcinoma. HC indicates healthy control. At-risk indicates noncancerous liver diseases, including cirrhosis and hepatitis. HBV indicates hepatitis B virus, HCV indicates hepatitis C virus, AFP indicates alpha fetoprotein, and BCLC stage indicates Barcelona Clinic Liver Cancer.

**Table 2:** Performances of plasma SEPT9 methylation (mSEPT9) and serum AFP in the diagnosis of hepatocellular carcinoma (HCC) or early-stage HCC.

Cut-off value		AUC		Sensitivity (%)	Specificity (%)	PPV (%)	NPV (%)	Accuracy (%)
		Value	95% CI					
HCC vs. HC								
mSEPT9	CT < 41	0.961	0.933-0.989	82.7	96.0	92.5	90.3	91.0
AFP	> 20 ng/mL	0.881	0.833-0.928	57.7	98.3	95.2	79.5	83.1
SEPT9+AFP		0.986	0.974-0.999	91.3	94.8	91.3	94.8	93.5
Early-stage HCC vs. HC								
mSEPT9	CT < 41	0.946	0.907-0.985	76.7	96.0	88.9	90.8	90.3
AFP	>20 ng/mL	0.852	0.789-0.914	52.1	98.3	92.7	83.0	84.6
SEPT9+AFP		0.980	0.962-0.999	87.7	94.8	87.7	94.8	92.7
HCC vs. at-risk								
mSEPT9	CT < 41	0.843	0.787-0.899	82.7	83.2	84.3	81.4	82.9
AFP	>20 ng/mL	0.812	0.751-0.873	57.7	86.3	82.2	65.1	71.4
SEPT9+AFP		0.863	0.812-0.914	91.3	76.8	81.2	89.0	84.4
Early-stage HCC vs. at-risk								
mSEPT9	CT < 41	0.799	0.73-0.868	76.7	83.2	77.8	82.3	80.4
AFP	>20 ng/mL	0.779	0.707-0.852	52.1	86.3	74.5	70.1	71.4
SEPT9+AFP		0.817	0.753-0.881	87.7	76.8	74.4	89.0	81.5

HCC indicates hepatocellular carcinoma. HC indicates healthy control. Early-stage HCC represents patients with early-stage hepatocellular carcinoma. At-risk indicates noncancerous liver diseases, including cirrhosis and hepatitis. AFP indicates alpha fetoprotein. CT indicates cycle threshold. AUC represents area under curve. CI indicates confidence interval. PPV indicates positive predictive value. NPV means negative predictive value.

## Data Availability

The data used to support the findings of this study are available from the corresponding author upon request.

## Abbreviations

*SEPT9*: *Septin9*
HCC: Hepatocellular carcinoma
HC: Healthy control
At-risk: Noncancerous liver diseases, including cirrhosis and hepatitis
HBV: Hepatitis B virus
HCV: Hepatitis C virus
AFP: Alpha fetoprotein
BCLC: Barcelona Clinic Liver Cancer
CT: Cycle threshold.

## Supplementary Materials

Supplementary Materials Figure S1: the plasma SEPT9 methylation (mSEPT9) level was analyzed by using the Wilcoxon-Mann-Whitney test. According to Donald's study (1), when standard deviations (SD) of two samples are unequal, a *t* test may have better type-1 error control than nonparametric alternatives, such as the Wilcoxon-Mann-Whitney test. In this case, the standard deviations of these groups are not equal, so we show the result of *t* test in text. Table S1: methylation-specific fluorescence quantitative PCR analysis of plasma *SEPT9* (mSEPT9). Table S2: correlation between SEPT9 promoter methylation and the clinicopathological parameters in patients with HCC.
