# Supplementary material for: SEPT9 Gene Methylation as a Noninvasive Marker for Hepatocellular Carcinoma
Source: Dis Markers. 2020 Oct 29;2020:6289063. doi: 10.1155/2020/6289063 (PMC7647768; doi:10.1155/2020/6289063)
Supplement: Supplementary Materials — Figure S1: the plasma SEPT9 methylation (mSEPT9) level was analyzed by using the Wilcoxon-Mann-Whitney test. According to Donald's study (1), when standard deviations (SD) of two samples are unequal, a t test may have better type-1 error control than nonparametric alternatives, such as the Wilcoxon-Mann-Whitney test. In this case, the standard deviations of these groups are not equal, so we show the result of t test in text. Table S1: methylation-specific fluorescence quantitative PCR analysis of plasma SEPT9 (mSEPT9). Table S2: correlation between SEPT9 promoter methylation and the clinicopathological parameters in patients with HCC. [file 6289063.f1.docx]

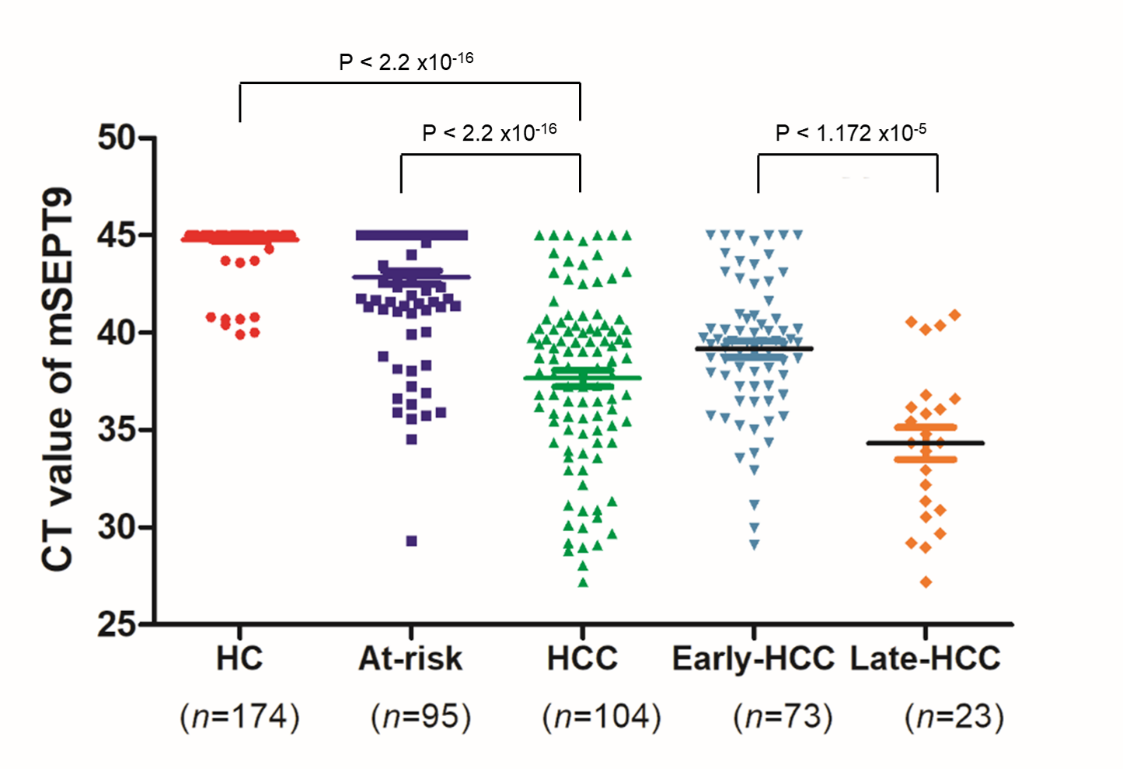


Supplementary Figure S1. The plasma SEPT9 methylation (mSEPT9) level were analyzed by using Wilcoxon-Mann-Whitney test. According to Donald’s study (1), when standard deviations (SD) of two samples are unequal, a t-test may have better type-1 error control than non-parametric alternatives, such as Wilcoxon-Mann-Whitney test. In this case the standard deviations of these groups are not equal, so we show the result of t-test in text.

1. Donald W. Zimmerman. Invalidation of Parametric and Nonparametric Statistical Tests by Concurrent Violation of Two Assumptions. *The Journal of Experimental Education* 1998, 67(1): 55-68.

Supplementary Table S1: Methylation-specific fluorescence quantitative PCR analysis of plasma *SEPT9* (mSEPT9).

|  | n | mSEPT9 | | |
| --- | --- | --- | --- | --- |
|  |  | mean CT ± SD | Range | *p* value |
| HCC | 104 | 37.7 ± 4.4 | 27.2 - 45 |  |
| At-risk disease | 95 | 42.8 ± 3.5 | 29.4 - 45 | < 0.001 |
| Healthy controls | 174 | 44.8 ± 0.9 | 39.9 - 45 | < 0.001 |

HCC indicates hepatocellular carcinoma. At-risk disease indicates non-cancerous liver diseases including cirrhosis and hepatitis. CT indicates cycle threshold. SD indicates standard deviation.

Supplementary Table S2: Correlation between *SEPT9* promoter methylation and clinicopathological parameters in patients with HCC.

|  | N | mSEPT9 | | χ^2^ | p value |
| --- | --- | --- | --- | --- | --- |
|  |  | Positive (%) | Negative (%) |  |  |
| Gender |  |  |  |  |  |
| Male | 83 | 70 (84.3) | 13 (15.7) | 0.777 | 0.378 |
| Female | 21 | 16 (76.2) | 5 (23.8) |  |  |
| Age (years) |  |  |  |  |  |
| 0-50 | 33 | 20 (60.6) | 12 (36.4) | 9.408 | 0.002 |
| >50 | 71 | 65 (91.5) | 6 (8.5) |  |  |
| AFP (ng/mL) |  |  |  |  |  |
| 0-20 | 44 | 35 (79.5) | 9 (20.5) | 0.528 | 0.468 |
| > 20 | 60 | 51 (85.0) | 9 (15.0) |  |  |
| HBeAg |  |  |  |  |  |
| Postive | 89 | 72 (80.9) | 17 (19.1) | 1.387 | 0.239 |
| Negative | 15 | 14 (93.3) | 1 (6.7) |  |  |
| BCLC stage |  |  |  |  |  |
| A | 46 | 33 (71.7) | 13 (28.3) | 8.916 | 0.030 |
| B | 27 | 23 (85.2) | 4 (14.8) |  |  |
| C/D | 23 | 23 (100.0) | 0 (0.0) |  |  |
| Unknown | 8 | 7 (87.5) | 1 (12.5) |  |  |

HCC indicates hepatocellular carcinoma. AFP indicates alpha fetoprotein, and BCLC stage indicates the Barcelona Clinic Liver Cancer stages.
